# Universal (meta-)logical reasoning: The Wise Men Puzzle (Isabelle/HOL dataset)

**DOI:** 10.1016/j.dib.2019.103823

**Published:** 2019-03-20

**Authors:** Christoph Benzmüller

**Affiliations:** a Freie Universität Berlin, Germany; b University of Luxembourg, Luxembourg

**Keywords:** Universal logical reasoning, Automated theorem proving, Higher-order logic

## Abstract

The authors universal (meta-)logical reasoning approach is demonstrated and assessed with a prominent riddle in epistemic reasoning: the Wise Men Puzzle. The presented solution puts a particular emphasis on the adequate modeling of common knowledge and it illustrates the elegance and the practical relevance of the shallow semantical embedding approach when utilized within modern proof assistant systems such as Isabelle/HOL. The contributed dataset provides supporting evidence for claims made in the article “Universal (meta-)logical reasoning: Recent successes” (Benzmüller, 2019).

**Table:** Specifications table

Subject area	*Computer Science*
More specific subject area	*Artificial intelligence, knowledge representation and reasoning*
Type of data	*Figures, .thy file (Isabelle/HOL syntax), readable (pdf) view of data*
How data was acquired	*The data was acquired through manual encoding of the Wise Men Puzzle in a higher-order multimodal logic that has been semantically embedded in the Isabelle/HOL proof assistant system.*
Data format	*Processed and analyzed data (The data is provided in the syntax format of the Isabelle/HOL proof assistant, which has been used to process, analyze and verify it; Isabelle/HOL is freely available at*https://isabelle.in.tum.de*)*
Experimental factors	*The data was manually constructed in order to empirically assess the expressivity and proof automation capabilities of Isabelle/HOL in epistemic reasoning when utilizing the shallow semantical embedding approach.*
Experimental features	*The shallow semantical embedding approach is confirmed as a suitable means to automate reasoning even in challenging (combinations of) non-classical logics.*
Data source location	*Berlin, Germany, Freie Universität Berlin, Institute of Computer Science*
Data accessibility	*Data is with this article*
Related research article	*C. Benzmüller, Universal (meta-)logical reasoning: Recent successes, Science of Computer Programming 172:48-62 (2019).*https://doi.org/10.1016/j.scico.2018.10.008*[1]*

**Table:** 

**Value of the data** • The contributed data in this article includes a reusable shallow semantical embedding of higher-order multimodal logic in HOL [2], interpretable in the Isabelle/HOL proof assistant system [4]. • The contributed higher-order multimodal has applications, among others, in computer science, artificial intelligence and metaphysics (concrete examples are outlined in the related research article). It can be reused, independent of the encoding of the Wise Men Puzzle, as a starting point for a wide range of formalization projects in these areas. Interested researchers only need to download the provided data file and include it in their formalization projects. • The contributed data also includes an encoding of the Wise Men Puzzle [6] in higher-order multimodal logic. This logic riddle constitutes a prominent benchmark example in the area of knowledge representation and reasoning. It can be reused as a starting point for the encoding and automated solution of similar challenge problems in epistemic reasoning (including other prominent logic riddles). Interested researchers only need to download the provided data file and include it in their formalization projects. • The presented encoding of the logic riddle puts a particular emphasis on the adequate modeling of common knowledge, which is defined here (following the suggestions of Sergot [5]) as the transitive closure of mutual knowledge of a group of agents. The agents mutual knowledge in turn is modeled as the union of the individual knowledge of the three agents (and their knowledge is encoded by using indexed box operators in modal logic KT45). This union, however, does not constitute a proper KT45 knowledge operator, since it is lacking a relevant transitivity property: if the group of agents A knows P, then this group A knows that it knows P; by taking the transitive closure of the agents mutual knowledge the issue is solved. • The adequate encoding of the notion of transitive closure poses a core challenge for knowledge representation frameworks; we here present a short and elegant solution in HOL (a single line of code, cf. Fig. 1). **Fig. 1:** 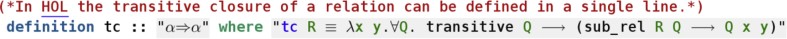 Higher-order definition of the transitive closure of a relation; encoding in Isabelle/HOL. • The data can be reused and further extended by researchers in various other areas, including artificial intelligence (knowledge representation and reasoning), computer science and metaphysics.

## 1 Data

**Fig. 2:**
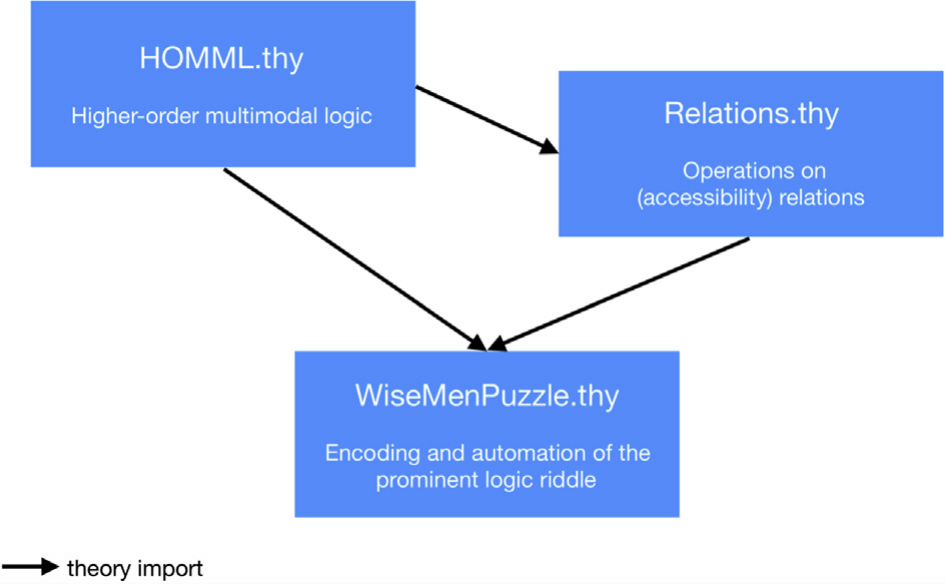
Workflow of the data interaction (import structure of the theory files); HOMML.thy is reusable independent of the other files.

The data is provided in form of three Isabelle/HOL source files (HOMML.thy, Relations.thy and WiseMenPuzzle.thy), which are bundled together in a single zip-file; the theory import dependencies of these files is depicted in Fig. 2.

HOMML.thy contains an encoding of higher-order multimodal logic K in classical higher-order logic following the authors shallow semantical embedding approach. Constant domain quantifiers are provided for all types in the HOL type hierarchy. The notion of logic combination adopted is that of a logic fusion [3]. The encoding supports both a local and global notion of modal validity (and logical consequence).

Relations.thy contains some basic and useful properties and operations on relations. Example properties are reflexivity, symmetry, transitivity and euclideaness; example operations are the union and intersection of relations. Most importantly, however, Relations.thy provides a higher-order definition for the transitive closure of a relation (cf. Fig. 1). This definition is utilized in file WiseMenPuzzle.thy to provide an adequate notion of common knowledge (of a group of agents).

WiseMenPuzzle.thy, which imports the two other files, contains the encoding of the Wise Men Puzzle. Utilizing the basic concepts from HOMML.thy, three indexed KT45 modal box operators are introduced in order to model the individual knowledge of the three agents in the given puzzle scenario (the introduction of further box operators is straightforward). Another indexed KT45 modal box operator is then defined to capture the common knowledge of these three agents (the modeling thus closely follows the suggestions of Sergot [5]).

File WiseMenPuzzle.thy also demonstrates how the logic riddle is solved automatically by the automated theorem provers integrated with Isabelle/HOL.

The Isabelle/HOL system is needed to properly interpret and verify the provided dataset; it can be obtained from https://isabelle.in.tum.de.

## 2 Experimental design, materials, and methods

The data was acquired through manual encoding of the problem in the Isabelle/HOL [4] proof assistant system. The motivation has been to empirically assess the expressivity and automated reasoning capabilities of Isabelle/HOL (with its various integrated automated reasoning tools) in epistemic reasoning when utilizing the shallow semantical embedding approach [2]. The encoding of the data was conducted in form of dialog between the author and the proof assistant. In this process the author provided type declarations, definitions and abbreviations, axiom postulates, lemmas and theorems. The proof assistant constantly monitored these activities, automatically analyzed the input, and offered permanent feedback. In addition to syntax checks and type checking, this also included the automated formal assessment of the conjectured lemmas and theorems with automated theorem provers. Moreover, the consistency of the provided premises was permanently monitored by model finding tools. The final result of the encoding process are the formally verified data documents as provided with this data article.

The automated assessment of the data in the described process was supported by a range of automated reasoning tools that are integrated with the Isabelle/HOL proof assistant via its Sledgehammer tool (cf. [4] for further details and references). This includes state-of-the-art first-order automated theorem provers (such as prover E, Vampire and SPASS), higher-order automated theorem provers (such as LEO-II and Satallax), satisfiability modulo solvers (such as CVC4 and Z3), and the model and countermodel finders Nitpick and Nunchaku. The feedback offered by these systems during the data encoding process was guiding the continuous emendations of the data until a fully verified data document was finally obtained. Fully verified in this context means that all proofs that were automatically constructed during the data encoding process have been reproduced and rechecked without failure in Isabelle/HOL's trusted kernel (a small set of trusted proof rules). Whenever the provided data files are read again by the Isabelle/HOL system the described data verification process is repeated.
