# Supplementary material for: Universal (meta-)logical reasoning: The Wise Men Puzzle (Isabelle/HOL dataset)
Source: Data Brief. 2019 Mar 20;24:103823. doi: 10.1016/j.dib.2019.103823 (PMC6468147; doi:10.1016/j.dib.2019.103823)
Supplement: Supplementary file 2 [file mmc2.zip › WiseMenPuzzle/DataInReadableForm.pdf]

Isabelle2018/HOL - HOMML.thy

```
theory HOMML imports Main                             (* By Christoph Benzmüller, 2018 *)

begin (*An Embedding of Higher-Order Multi-Modal Logic (HOMML) in HOL.*)

  typedecl i (*Type of possible worlds.*) typedecl μ (*Type of individuals.*)
  type_synonym σ="(i⇒bool)" (*Type of world depended formulas (truth sets).*)
  type_synonym α="(i⇒i⇒bool)" (*Type of accessibility relations between worlds.*)

 (*Lifted HOMML connectives: they operate on world depended formulas (truth sets).*)
  definition mtop :: "σ" ("⊤") where "⊤ ≡ λw. True"
  definition mbot :: "σ" ("⊥") where "⊥ ≡ λw. False"
  definition mneg :: "σ⇒σ" ("¬_"[52]53)  where "¬φ ≡ λw. ¬φ(w)"
  definition mand :: "σ⇒σ⇒σ" (infixr"∧"51) where "φ∧ψ ≡ λw. φ(w)∧ψ(w)"
  definition mor  :: "σ⇒σ⇒σ" (infixr"∨"50) where "φ∨ψ ≡ λw. φ(w)∨ψ(w)"
  definition mimp :: "σ⇒σ⇒σ" (infixr"→"49) where "φ→ψ ≡ λw. φ(w)⟶ψ(w)"
  definition mequ :: "σ⇒σ⇒σ" (infixr"↔"48) where "φ↔ψ ≡ λw. φ(w)⟷ψ(w)"
  definition mall :: "('a⇒σ)⇒σ" ("∀") where "∀Φ ≡ λw.∀x. Φ(x)(w)"
  definition mallB:: "('a⇒σ)⇒σ" (binder"∀"[8]9) where "∀x. φ(x) ≡ ∀φ"
  definition mexi :: "('a⇒σ)⇒σ" ("∃") where "∃Φ ≡ λw.∃x. Φ(x)(w)"
  definition mexiB:: "('a⇒σ)⇒σ" (binder"∃"[8]9) where "∃x. φ(x) ≡ ∃φ"
  definition mbox :: "α⇒σ⇒σ" ("□_ _") where "□ r φ ≡ (λw. ∀v. r w v ⟶ φ v)"
  definition mdia :: "α⇒σ⇒σ" ("◇_ _") where "◇ r φ ≡ (λw. ∃v. r w v ∧ φ v)"

 (*Global and local validity of lifted formulas*)
  definition global_valid :: "σ ⇒ bool" ("⌊_⌋"[7]8) where "⌊φ⌋ ≡ ∀w. φ w"
  consts cw :: i (*Current world; uninterpreted constant of type i*)
  definition local_valid :: "σ ⇒ bool" ("⌊_⌋cw"[9]10) where "⌊φ⌋cw ≡ φ cw"

 (*Introducing "Defs" as the set of the above definitions; useful for convenient unfolding.*)
 named_theorems Defs declare mtop_def[Defs] mbot_def[Defs] mneg_def[Defs] mand_def[Defs]
  mor_def[Defs] mimp_def[Defs] mequ_def[Defs] mall_def[Defs] mallB_def[Defs] mexi_def[Defs]
  mexiB_def[Defs] mbox_def[Defs] mdia_def[Defs] global_valid_def[Defs] local_valid_def[Defs]
end
```

Isabelle2018/HOL - Relations.thy

```
theory Relations imports HOMML                    (* By Christoph Benzmüller, 2018 *)
begin
 (*Some useful properties and operations on (accessibility) relations*)
  definition reflexive :: "α⇒bool" where "reflexive R ≡ ∀x. R x x"
  definition symmetric :: "α⇒bool" where "symmetric R ≡ ∀x y. R x y ⟶ R y x"
  definition transitive :: "α⇒bool" where "transitive R ≡ ∀x y z. R x y ∧ R y z ⟶ R x z"
  definition euclidean :: "α⇒bool" where "euclidean R ≡ ∀x y z. R x y ∧ R x z ⟶ R y z"
  definition intersection_rel :: "α⇒α⇒α" where "intersection_rel R Q ≡ λu v. R u v ∧ Q u v"
  definition union_rel :: "α⇒α⇒α" where "union_rel R Q ≡ λu v. R u v ∨ Q u v"
  definition sub_rel :: "α⇒α⇒bool" where "sub_rel R Q ≡ ∀u v. R u v ⟶ Q u v"
  definition inverse_rel :: "α⇒α" where "inverse_rel R ≡ λu v. R v u"

 (*In HOL the transitive closure of a relation can be defined in a single line.*)
  definition tc :: "α⇒α" where "tc R ≡ λx y.∀Q. transitive Q ⟶ (sub_rel R Q ⟶ Q x y)"

 (*Adding the above definitions to the set of definitions Defs.*)
 declare reflexive_def[Defs] symmetric_def[Defs] transitive_def[Defs] euclidean_def[Defs]
   intersection_rel_def[Defs] union_rel_def[Defs] sub_rel_def[Defs] inverse_rel_def[Defs]

 (*Some useful lemmata.*)
  lemma trans_tc: "transitive (tc R)" unfolding Defs tc_def by metis
  lemma trans_inv_tc: "transitive (inverse_rel (tc R))" unfolding Defs tc_def by metis
  lemma sub_rel_tc: "symmetric R ⟶ (sub_rel R (inverse_rel (tc R)))"
    unfolding Defs tc_def by metis
  lemma sub_rel_tc_tc: "symmetric R ⟶ (sub_rel (tc R) (inverse_rel (tc R)))"
    using sub_rel_def sub_rel_tc tc_def trans_inv_tc by fastforce
  lemma symm_tc: "symmetric R ⟶ symmetric (tc R)"  sledgehammer [verbose]  nitpick
    using inverse_rel_def sub_rel_def sub_rel_tc_tc symmetric_def by auto
end
```

Isabelle2018/HOL - WiseMenPuzzle.thy

```
theory WiseMenPuzzle imports HOMML Relations                   (* By Christoph Benzmüller, 2018 *)
begin
  consts a::"α" b::"α" c::"α"  (*Wise men modeled as accessibility relations.*)
 (*Reflexivity, transitivity and euclideaness is postulated for these relations.*)
  axiomatization where
   knowl_abc: "(x = a ∨ x = b ∨ x = c) ⟹ (reflexive x ∧ transitive x ∧ euclidean x)"
 (*Eabc φ stands for "Everyone in group {a,b,c} knows φ".*)
  definition Eabc where "Eabc ≡ union_rel (union_rel a b) c"
 (*Cabc φ stands for "The common knowledge of group {a,b,c}".*)
  definition Cabc :: "i⇒i⇒bool" where "Cabc ≡ tc Eabc"
 (*Cabc is reflexive, transitive and euclidean; i.e. it is a knowledge operator.*)
  lemma refl_Cabc: "reflexive Cabc" using Cabc_def Eabc_def knowl_abc Defs tc_def by smt
  lemma symm_C_abc: "symmetric Cabc" using Cabc_def Eabc_def knowl_abc symm_tc Defs by smt
  lemma eucl_Cabc: "euclidean Cabc" using Cabc_def Eabc_def knowl_abc symm_C_abc Defs tc_def by smt
 (*We are now ready to model and automate the wise men puzzle.*)
  consts ws :: "α⇒σ" (*ws a: a has a white spot.*) wise :: "α⇒bool" (*wise a: a is a wise man.*)
  axiomatization where
   A0: "wise a ∧ wise b ∧ wise c" (*a, b and c are wise men.*) and
   (*Common knowledge: at least one of a, b and c has a white spot *)
   A1: "⌊□Cabc (ws a ∨ ws b ∨ ws c)⌋" and
   (*Common knowledge: if x has a white spot then y can see this (and hence know this).*)
   A2: "wise x ∧ wise y ∧ x ≠ y ⟹ ⌊□Cabc (ws x ⟶ □y (ws x))⌋" and
   (*Common knowledge: if x not has a white spot then y can see this (hence know this) *)
   A3: "wise x ∧ wise y ∧ x ≠ y ⟹ ⌊□Cabc (¬(ws x) ⟶ □y (¬(ws x)))⌋" and
   (*Common knowledge: a does not know whether he has a white spot.*)
   A4: "⌊(□Cabc (¬(□a (ws a))))⌋"  and
   (*Common knowledge: b does not know whether he has a white spot.*)
   A5: "⌊(□Cabc ¬(□b (ws b)))⌋"
  theorem  (*c knows he has a white spot.*)
   T1: "⌊(□c (ws c))⌋" using A0 A1 A3 A4 A5 refl_Cabc unfolding Defs sledgehammer()
    by metis
lemma True nitpick [satisfy,user_axioms,expect=genuine,show_all] oops (*Consistency confirmed*)
end
```
